# Understanding factors affecting implementation success and sustainability of a comprehensive prevention program for cardiovascular disease in primary health care: a qualitative process evaluation study combining RE-AIM and CFIR

**DOI:** 10.1017/S1463423623000063

**Published:** 2023-03-08

**Authors:** Naomi Aerts, Kathleen Van Royen, Peter Van Bogaert, Lieve Peremans, Hilde Bastiaens

**Affiliations:** 1 Department of Family Medicine and Population Health, Faculty of Medicine and Health Sciences, University of Antwerp, Antwerp, Belgium; 2 Department of Nursing and Midwifery, Faculty of Medicine and Health Sciences, University of Antwerp, Antwerp, Belgium; 3 Department of Communication Studies, Faculty of Social Sciences, University of Antwerp, Antwerp, Belgium

**Keywords:** action research, cardiovascular diseases, implementation science, primary health care, primary prevention, process evaluation

## Abstract

**Aim:**

Our aim was to evaluate the implementation process of a comprehensive cardiovascular disease prevention program in general practice, to enhance understanding of influencing factors to implementation success and sustainability, and to learn how to overcome barriers.

**Background:**

Cardiovascular disease and its risk factors are the world’s leading cause of mortality, yet can be prevented by addressing unhealthy lifestyle behavior. Nevertheless, the transition toward a prevention-oriented primary health care remains limited. A better understanding of factors facilitating or hindering implementation success and sustainability of prevention programs, and how barriers may be addressed, is needed. This work is part of Horizon 2020 project ‘SPICES’, which aims to implement validated preventive interventions in vulnerable populations.

**Methods:**

We conducted a qualitative process evaluation with participatory action research approach of implementation in five general practices. Data were collected through 38 semi-structured individual and small group interviews with seven physicians, 11 nurses, one manager and one nursing assistant, conducted before, during, and after the implementation period. We applied adaptive framework analysis guided by RE-AIM Qualitative Evaluation for Systematic Translation (RE-AIM QuEST) and Consolidated Framework for Implementation Research (CFIR).

**Findings:**

Multiple facilitators and barriers affected reach of vulnerable target populations: adoption by primary health care providers, implementation and fidelity and intention to maintain the program into routine practice. In addition, our study revealed concrete actions, linked to implementation strategies, that can be undertaken to address identified barriers. Prioritization of prevention in general practice vision, ownership, and shared responsibility of all team members, compatibility with existing work processes and systems, expanding nurse’s roles and upskilling competence profiles, supportive financial and regulatory frameworks, and a strong community – health care link are crucial to increase implementation success and long-term maintenance of prevention programs. COVID-19 was a major barrier to the implementation. RE-AIM QuEST, CFIR, and participatory strategies are useful to guide implementation of prevention programs in primary health care.

## Background

Cardiovascular diseases are the world’s leading cause of mortality with around 18,6 million deaths in 2019, representing 32% of global mortality (Nichols *et al.*, 2014; World Health Organization, 2021) and 393 million disability-adjusted life years (Townsend *et al*., 2016; Roth *et al.*, 2020). The financial burden of cardiovascular disease and its risk factors on society and the health care system is substantial (Budig & Harding, 2021). The burden is highest among individuals with lower socioeconomic status (Min *et al*., 2017; GBD 2017 Risk Factor Collaborators, 2018). Current evidence underpins the association between low socioeconomic status and cardiovascular disease, its risk factors, and unhealthy lifestyle behaviors (Sommer *et al*., 2015).

The World Health Organization estimates that nearly 75% of premature deaths are preventable (World Health Organization, 2021). Healthy lifestyle practices including smoking cessation, healthy diets, physical activity, and alcohol reduction are important in the prevention of cardiovascular disease and its modifiable risk factors such as hypertension, (pre-) diabetes, dys- and hyperlipidaemia, overweight, and obesity (Yusuf *et al*., 2020). Current evidence demonstrates numerous strategies to reduce cardiovascular disease risk with strong consensus on the importance of raising awareness of risk factors and on the impact of lifestyle on health outcomes (Stewart *et al*., 2017; Díaz-Gutiérrez *et al*., 2019; Hassen *et al*., 2021). Clinical practice guidelines yet fail to consistently propose structured protocols to guide practitioners, and gaps in evidence are reported especially regarding strategies targeting vulnerable populations (Odorico *et al*., 2019; Aerts *et al*., 2021). Consequently, people with low socioeconomic status tend to benefit less from preventive care including lifestyle interventions (Coupe *et al*., 2018; Rosengren *et al.*, 2019).

A critical research–practice gap on actual implementation of structured preventive interventions indeed remains. Studies show poor achievement of guideline-recommended cardiovascular disease prevention targets (Kotseva *et al.*, 2019, Kotseva *et al*., 2020). As such, there is an urgent need to further develop and implement interventions and strategies for detection and management of risk factors, in the general population as well as in vulnerable subpopulations. Horizon 2020 funded ‘Scaling-up Packages of Interventions for cardiovascular disease prevention in selected sites in Europe and sub-Saharan Africa’ (SPICES) project was established with the aim to implement evidence-based interventions for primary prevention in the population, including vulnerable groups, in low-, middle-, and high-income countries such as Belgium, where this study was carried out.

In Belgium, as in other high-income countries, prevention is primarily performed in primary health care, yet health systems fail to provide systematic support for all aspects of prevention. General practice plays a critical role in prevention and can be valuable in addressing socioeconomic health differences due to frequent contact with a large and often diverse target populations (Si *et al*., 2014). However, prevention-orientated services are not systematically provided in Belgian general practice. Clinical practice guidelines report various interprofessional collaboration models, including role expansion and task delegation in primary health care (Aerts *et al.*, 2021). Integrated care delivered by physisiancs and nurses in general practice brings the opportunity to increase quality and accessibility of preventive care (Philips *et al*., 2015; Waller *et al*., 2016; Matthys *et al.*, 2017; Srivarathan *et al*., 2019; Aerts *et al*., 2020). However, little is known about how to implement validated preventive interventions in a specific real-life context of general practice and to which extent new interdisciplinary, collaborative forms can enhance their uptake.

This study aimed to explore how a comprehensive cardiovascular disease prevention program can be implemented in general practice in a high-income country as Belgium. The aim of this study is to understand the influencing factors and facilitators for a successful implementation and sustainability and to learn how to overcome barriers. Through insight into the implementer’s experiences with the process and in the critical role of nurses, these findings provide guidance for research and practice groups that wish to scale up validated interventions for the prevention of cardiovascular disease in primary health care.

## Methods

### Study design and frameworks

This paper reports the qualitative process evaluation of an implementation carried out from an empowering, collaborative, and change-oriented research perspective and framed within the transformative paradigm (Creswell & Clark, 2011; Jackson *et al.*, 2018). We applied principles of participatory action research (Koshy *et al.*, 2011; Mortelmans, 2013) to guide the implementation process, meaning that key stakeholders were involved in the co-creation, critical reflection and dynamic, context-specific tailoring of the program throughout the different stages of our implementation study.

The expanded RE-AIM Qualitative Evaluation for Systematic Translation (RE-AIM QuEST) framework, as proposed by Forman *et al*. (2017), guided our formative process evaluation to identify real-time implementation barriers and explain how the context may influence sustainability and scale up to other settings (Glasgow *et al.*, 1999; Forman *et al.*, 2017). The complexity of the implementation context supports the use of qualitative methods as proposed by Holtrop *et al*., as they provide insight into ‘why and how’ our implementation process led to certain results, but it also encouraged collaborative stakeholder engagement (Holtrop *et al*., 2018). In this paper, we report on the qualitative evaluation of RE-AIM dimensions ‘reach’ (participation of the target population), ‘adoption’ (participation of general practices and implementers), ‘implementation’ (including fidelity), and ‘maintenance’ (of the intervention). The quantitative evaluation will be reported elsewhere, as will the qualitative evaluation of RE-AIM dimension ‘effectiveness’ from participants’ perspective.

The Consolidated Framework for Implementation Research (CFIR) (Damschroder *et al*., 2009; Nilsen, 2015; Nilsen & Bernhardsson, 2019), a comprehensive framework consisting of constructs associated with successful implementation, was applied to further gain understanding in implementation determinants influencing the RE-AIM dimensions. The complementary use of the RE-AIM evaluation framework and the CFIR determinant framework was previously demonstrated by King *et al.* (2020).

### Description of the intervention and target population

The evidence-based SPICES program combines principles of Prochaska’s and Diclemente transtheoretical model (Prochaska *et al*., 2015), self-determination theory (Deci & Ryan, 2012), motivational interviewing (Miller & Rollnick, 2012), and brief action planning (Gutnick *et al*., 2014) and consists of two major components. The first ‘profiling’ component included cardiovascular disease risk stratification and communication applying the non-laboratory INTERHEART modifiable risk score (Yusuf *et al.*, 2004). We selected this tool because of its practical usability by nurses without needing supervision or intervention of physicians. The tool uses simple questions related to lifestyle behavior risk and a waist–hip circumference measurement to allocate ones individual risk to a high-, intermediate-, or low-risk category. The profiling component was carried out by the nurse during a single session with an average duration of 20 min. The nurses used risk communication and motivational interviewing techniques to discuss the result and to initiate the appropriate follow-up trajectory. Participants either received a very brief advice on how to maintain a healthy lifestyle (low-risk score), or information on appropriate follow-up trajectories based on their individual risk score (intermediate to high-risk score).

The second ‘coaching’ component, consisted of multi-lifestyle-behavior change counseling for those at medium to high risk with one-year follow-up, spread in 10 sessions following a set interval and with a duration of approximately 30 to 45 min. The coaching sessions were focused on Dietary Approaches to Stop Hypertension (DASH) diet (Guo *et al.*, 2021; Lari *et al.*, 2021); combined aerobic training or aerobic and resistance physical activity; smoking cessation, and comprised behavior change techniques such as motivational interviewing, goal-setting, action-planning, and problem-solving. The intervention was delivered face to face in individual sessions. We targeted vulnerable communities using the principles of proportionate universalism (Marmot, 2012), focusing on (sub-)population-level vulnerability rather than on individual level; thus, the intended group was reached on the level of study setting. On individual level, we targeted adults between 40 and 75 years old who were not diagnosed with cardiovascular disease. People with known diabetes were excluded since they are already included in an existing care protocol including lifestyle guidance. The strategies that were used to inform, invite, and engage the target population differed in each setting, for example, passive invitation through posters; personal invitation during a contact; email or telephone invitation. Prior to the implementation, all relevant implementers received training on techniques for participant recruitment, risk profiling, and communication and lifestyle behaviour change counseling. They were also granted access to the project tools designed to support all intervention components. Both training and tools were developed by the research group in collaboration with experts in the field. A comprehensive description of the intervention, based on the Template for Intervention Description and Replication (TIDieR) checklist (Hoffmann *et al.*, 2014), is provided as supplementary material 1, and its development will be fully described elsewhere.

### Study setting and implementation

The intervention was rolled out in general practices in the Dutch-speaking Antwerp region. Multidisciplinary general practices with nurse integration were eligible for inclusion. General practices were eligible if they served a diverse population including vulnerable people with low socioeconomic status and/or if they were located in vulnerable city districts in Antwerp. Districts’ vulnerability was identified based on socioeconomic health deprivation index, limited access to primary health care, and density of households with social support. Twenty eligible practices were contacted by email or telephone, five of which were willing to participate initially (setting characteristics are summarized in Table 1). Two practices (Practice D and practice E) decided to stop participation before actual implementation took place. The three remaining settings (Practice A, B, and C) completed all implementation phases and fully implemented all intervention components. General practices did not receive any financial incentive or compensation for study participation since this would hamper sustainability of the implementation beyond the study period. They were encouraged to embed project-related activities in their regular financial system as outlined in Table 1.

**Table 1.** Characteristics and description of contextual factors and implementation details of included settings

		Practice A	Practice B	Practice C	Practice D	Practice E
Setting characteristics	Type	Group, multidisciplinary	Group, multidisciplinary	Group, multidisciplinary	Group, multidisciplinary	Group, multidisciplinary
	Geographical location	Inner city	Inner city	Urban	Urban	Rural
	Financial structure	Capitation system	Capitation system	Capitation system	Capitation system	Capitation system
Population characteristics	# Patients	4539	4027	3217	3217	2100
	# Age 40–75	1491	1296	1358	^*^	1042
	# Increased reimbursement	1670	765	670	^*^	633
Implementation roadmap		PRE	PRE	PRE	PRE	PRE
		PER 1 > PER 2 > PER 3 > PER 4	PER 1 > PER 2 > PER 3	PER 1 > PER 2	EXIT	EXIT
		POST	POST	POST		
Key implementers	General practitioner	1	2	1	1	2
	Practice nurse	2	3	2	2	2
	Practice nurse assistant	0	0	1	0	0
	Practice manager	1	0	0	0	0
Participant reach	# Profiled	37	20	13	N/A	N/A
	# Started coaching	15	7	7	N/A	N/A
Pre-implementation contextual information	Vision and mission	Practice in transition: integrated interdisciplinary care; high-quality care; accessibility of care; holistic approach	Accessibility of care; supporting vulnerable population; holistic approach; interdisciplinary care; high-quality care; training- and research-oriented	Accessibility of care; empowering people for health; equal partnership and interdisciplinary care	Accessibility of care; interdisciplinary care; community link; prevention	Practice in transition: interdisciplinary team expansion and capitation system; empowering population; Prevention
	Preexisting community link	Current gap; planned team expansion with social worker; insufficient knowledge of community resources	Referral to physical activity on prescription; referral to external care partners; planning to focus on community-oriented care in future	Current gap; mainly internal follow-up; referral to physical activity on prescription; referral to external care partners	Link with local welfare organization; referral to external care partners; planning community-outreaching initiatives in future	Current gap; mainly internal follow-up
	Current practices for prevention	Focus on secondary prevention; lack of structural organization and integration of primary prevention protocols	Focus on secondary prevention; primary prevention of lower priority; clear care plan and lifestyle follow-up for diabetes; unsuccessful previous attempts to implement prevention protocols	Lack of structural organization and integration of primary prevention; lack of continuity on lifestyle advice; planning to introduce prevention consultation in future; existing prevention protocols are too complex	Ad hoc prevention consultations; clear care plan for diabetes; no structural focus on cardiovascular disease	Ad hoc prevention consultations; clear care plan for diabetes; no structural focus on cardiovascular disease; lifestyle trajectory in collaboration with multidisciplinary team
	Practice nurse integration level	Transition from instrumental toward more integrated, autonomous role; level of task delegation depends on individual physician; limited role in prevention	Transition from instrumental toward more integrated, autonomous role; integrated through protocol care in management of chronic diseases	Transition from instrumental toward more integrated, autonomous role; central role in planned prevention consultations	Combined instrumental tasks and integrated through autonomous consultations for prevention and follow-up of chronic diseases	Combined instrumental tasks and integrated through autonomous consultations for diabetes follow-up

Group practice: >2GPs.

* Missing data.

N/A: Not applicable.

A stepwise implementation of the intervention, developed in August 2019, was planned so that the key learnings and good practices could be scaled up from one setting to the next one. All five settings completed the *pre-implementation phase*, which included thorough context analysis, implementation planning, and preparation of intervention components and key implementers. In Practice A, implementation took off in September 2020; Practice B started in February 2021; and for Practice C this was in July 2021. Implementation in all settings ran until December 2021. Our stepwise approach implies a difference in duration of the *per-implementation* phase in each of the three implementation settings; a phase where every two to three months, we undertook reflective action research spirals, allowing the researchers and key implementers to continuously monitor the dynamic course of the implementation and to incorporate new understandings into the ongoing process. The *post-implementation phase*, which ran from January up till June 2022, was mainly focused on providing necessary key requirements to consolidate intervention components long term and to see how this can be scaled up to a broader context. The implementation ‘roadmap’ of each of the settings is incorporated in Table 1.

All members of the primary health care teams in each of the five included settings were considered ‘implementers’, since all of them were directly or indirectly involved in the implementation process. However, the most critical role was laid out for the nurse who carried out the intervention. The target population was approached and informed by their primary health care provider, and, if interested, they were invited to make an appointment with the nurse. In the included settings, nurses (and one nurse assistant) carried out all intervention components. In case of high-risk participants, a shared decision on the appropriate follow-up trajectory was made between nurse, physician, and participant. A total of 70 participants were profiled, 29 of which were enrolled in the coaching trajectory (Table 1).

A comprehensive analysis of the study context, including the needs and anticipated challenges to implementation, is available elsewhere (Aerts *et al.*, 2022).

### Data collection

Data collection for this process evaluation ran simultaneously to the implementation process in each setting and was completed by March 2022. Data collection primarily consisted of 38 individual or small group interviews conducted at various stages of the implementation process. Small group interviews usually consisted of two to three implementers from the same setting, providing insight into the team’s shared implementation experience through interaction. A total of 20 key implementers from the five included settings were interviewd. Key implementers were defined as implementers who were closely involved in the planning, coordination, and/or execution of the implementation and consisted of seven physicians, 11 nurses, one nursing assistant, and one practice manager. The interviews were conducted face to face when feasible, or online in video conferences depending on COVID-19-related government guidelines at the time, and each lasted between 30 and 90 min. Interviews were carried out by a team of five research assistants under the supervision of an experienced research team. All interviews were audio-recorded, and the interviewers took extensive notes during and immediately after the interviews. The interviews were transcribed as soon as possible afterward.

The main issues brought up during the interviews were regularly discussed with the larger group of implementers during their preexisting team meetings in the primary care practices. On its turn, this input was fed back to the researchers during other contact moments. This way, we ensured that the entire primary health care team in each setting was always challenged to reflection and their experience was also incorporated in our process evaluation. Additionally, we documented all implementation activities, progress, and all communications in a logbook of each setting. We kept meeting reports from all informal meetings with the implementers in order to further support thorough process mapping.

Semi-structured interview guides based on the CFIR and RE-AIM QuEST, tailored to the context and targeted implementers, were developed to answer our research questions related to each data collection phase (pre-, per-, and post-implementation) (see supplementary material 2). The topic guide included specific questions on each setting’s context, the implementation process, the facilitators and barriers to implementation of each component, adaptations that were needed, and factors influencing implementation sustainability. During this process evaluation, we also assessed the intervention components, the supporting project tools, and the implementation strategies used. In order to map the barriers and facilitators to adoption and to understand reasons for dropping out, exit interviews were also conducted with the practices that decided to drop out. We pilot-tested the interview guides and made refinements based on respondent’s feedback and researcher’s experience.

### Data analysis

We analyzed all interview transcripts and documents using adaptive framework analysis (Gale *et al*., 2013; Ward *et al*., 2013) based on RE-AIM and CFIR, ensuring the possibility to also integrate text fragments that could not be placed in rigid preexisting categories. An a priori codebook was created based on RE-AIM and CFIR domains and constructs. The analysis was guided by operationalization of the four target dimensions for this study (reach, adoption, implementation, and maintenance). These clear descriptions supported the coders’ process in assigning relevant text to one of the four dimensions. Furthermore, operational definitions of CFIR domains and constructs were tailored to the study to improve coder consistency (see supplementary material 3). In the first phase of the coding process, text fragments that represented one of the four dimensions were identified. The output of the first coding phase was reviewed within the larger team of researchers, and discrepancies were solved through team discussion until consensus was reached. In a second phase, all allocated text fragments per RE-AIM dimension were subjected to a more in-depth coding procedure with the goal to further structure the text into relevant CFIR domains and (sub-) constructs. The output of this second phase was also discussed and refined based on iterative reflection cycles of the research team. Once analysis of interview data was completed, we conducted a document analysis of logbooks and meeting report guided by the final codebook. This analysis was used for the purpose of data triangulation of our primary interview data. Our data analysis was supported by QSR NVivo software version 1.5.1. This paper is built up using the consolidated criteria for reporting qualitative research (COREQ) checklist (Tong *et al.*, 2007) and Standards for Reporting Implementation Studies (StaRI) statement (Pinnock *et al*., 2017) as guidance.

## Results

With Table 2, we provide a comprehensive summary of facilitators and barriers to reach, adoption, and implementation, structured around relevant CFIR domains and constructs. Key findings of our per- and post-implementation process evaluation are presented in the following paragraphs structured by the covered RE-AIM domains, reinforced by implementers’ quotes. Table 3 consists of some clear examples of how barriers that were encountered by the implementers were addressed during the cyclic participatory action research process. We hereby give an overview of the implementation strategies that were applied, adapted from (Powell *et al*., 2015; Waltz *et al*., 2015), together with associated concrete actions as taken by implementers and the project team.

**Table 2.** Barriers and facilitators to reach, adoption, and implementation; structured by CFIR domains and constructs

RE-AIM domain	CFIR domain	CFIR construct	Facilitator	Barrier
Reach	Intervention characteristics	Adaptability	The intervention fits the needs and preferences of the target population, and is adaptable further along the implementation process	
		Complexity		Intensity of coaching trajectory, regarding number, frequency and duration of sessions, is discouraging
	Outer setting	Target population needs and resources	Target population is open to and interested in learning more about the intervention	Prevention is not a priority in vulnerable populations due to invisibility of the (potential) cardiovascular disease risk and presence of other multilevel complex issues
				Lack of ownership over own health
			Positive expectations regarding potential health benefits of the intervention	Low health literacy including knowledge and skills on how to access primary care services and the intervention
			Favorable stage of change: Intrinsic motivation and willingness to (think about) changing behavior	
			Fit with need for social support and connectedness is appreciated	
		Variable factors		COVID-19 pandemic causes fear in target population of going to ‘contaminated environment’ and of unnecessarily burdening health care providers
	Inner setting	Structural characteristics	Low threshold financial system increases accessibility of care, including the intervention	
	Characteristics of implementers	Self-efficacy		Nurses’ low confidence in own competences affects reach results in consciously excluding/avoiding certain sub-populations (e.g., ‘difficult to change’)
		Other personal attributes	Nurses’ and physicians’ values of genuine interest and involvement in health and wellbeing of target population	
	Process	Engaging participants	Giving tailor-made information to target population, using supporting materials	Insufficient or inconsistent information during invitation to participate
			Empowering target population by respecting autonomy and ownership	
			Combining recruitment strategies with case finding	Systematically inviting target population by email, letter, telephone implicates high administrative burden and low response rates
			Taking personal approach in addressing and inviting target population	
		Executing	Critical role of physicians’ trust-based relationship with target population for active recruitment	Low relative priority for active recruitment in physicians
			Regular reminders for recruitment and use of supporting materials; for example information sheet in physicians’ and nurses’ offices	Low fidelity of planned recruitment strategies in physicians
Adoption	Intervention characteristics	Relative advantage	Opportunity to improve current prevention practices, or to introduce a prevention program, in a structured way with support from project team	
			Focus on cardiovascular disease with population-wide impact potential	
			Expected health gain in target population	
			Access to evidence-based project tools and supporting materials; for example profiling tool, lifestyle plan, training	
			Opportunity to explore and expand nursing roles	
		Adaptability	Flexibility of the intervention to be tailored to each specific setting’s needs, preferences and capacity	
		Complexity		Intensity of the intervention and level of engagement, including the research component-related burden (e.g., data collection)
		Cost		Estimated personnel cost, especially regarding intensity of nurse project activities
	Outer setting	External policies and incentives		Lack of appropriate legal and financial frameworks to support prevention in primary health care and collaboration with nurses in general practice
		Variable factors		COVID-19 poses a major burden general practice with very high workload and unpredictable impact on practice
	Inner setting	Structural characteristics	Multidisciplinary group practice capacity	Lack of structural collaboration among disciplines
		Implementation climate	Supportive leadership	Differing receptivity to the intervention among involved members of larger teams
			Strong need for improving and more systematically embedding prevention in general practice	
			Strong need to expand nursing roles	
			Compatibility of the intervention with practice vision and mission	Insufficient compatibility of some project tools with existing workflows and systems
		Readiness for implementation		Insufficient resources for new capacities; both time and financial
	Process	Engaging Implementers	Creating wide support within the team by involving all team members from earliest stages	
			At least one nurse and one physician willing to lead, support, and reinforce the implementation (internal implementation leaders and champions)	
		Planning		Ambiguous implementation plans and tasks in the earliest stages of the project
Implemen-tation	Intervention characteristics	Relative advantage	Training on behavior change techniques widely transferable to general practice	Initial training proposed by project team remains theoretical and lacks concrete applicability to practice
			Additional expert supervision session on behavior change counseling strongly increases competences and self-efficacy	
		Adaptability	Flexibility of the intervention components and implementation strategies allow necessary adaptations	
		Trialability	Aligning project targets with setting-specific feasibility; for example by limited and stepwise recruitment of participants	
		Complexity		Coaching component intensity and prescribed format hindering fidelity
				Behavior change counseling-related challenges; for example reaching behavior change in vulnerable participants, insufficient insight in ‘active ingredients’ for behavior change
		Design Quality and Packaging	Project tools including strong visuals and useful, informative, relevant elements; guiding and supporting behavior change counseling and facilitating activity planning and follow-up of participants; increasing feasibility and fidelity of intervention components	Project tools including complex and ambiguous elements; increasing time investment needed and hindering fidelity
			Attractive format and design	
	Outer setting	Target population needs and resources	‘Warm referral’ to community resources; including personal introduction and practical support from nurses’ trust-based relationship (built during coaching sessions)	Lack of active partnership and input from participants
				Financial barriers and need for trust-based relationships hindering the referral of participants to community resources
		Cosmopolitanism	Getting personally acquainted in building a network for gaining trust in care partners and defining (shared) responsibilities	Lack of a team member (e.g., social worker) with dedicated time to map and engage community resources to refer to
			Coaching component triggering implementers to purposefully build health care and welfare partnerships meeting participants’ needs	
		Variable factors		COVID-19-related workload and governmental measures posing major barriers to implementation and continuity of planning and performing project activities
	Inner setting	Structural characteristics	Financial system supporting prevention and collaboration with nurses	Discontinuity of team composition
		Networks and communications	Regular team meetings to discuss participant cases and implementation; increasing involvement, adoption and collaboration in team members; platform for raising concerns and actively solving problems	Lack of coordination and insufficient structural communication, hindering project follow-up
		Implementation climate	Delegation of cardiovascular disease prevention to the nurse; interdisciplinary collaboration fits within existing workflows and systems	
		Readiness for implementation		Inadequate resources for new capacities; limited time availability for implementers to perform project tasks
	Characteristics of implementers	Self-efficacy	Targeted training support and regular practice, increasing nurses’ self-confidence especially regarding the behavior change counseling (coaching) component	Lack of feedback on performance from participants and/or knowledgeable expert
			Sharing experiences with peers, adding to professional growth	Tension field of to what extent to rely on own capabilities and when to call in other expertise (health care/welfare partners, community resources)
			Visible results and progress regarding lifestyle, wellbeing and risk perception in participants, confirming nurses’ feeling of being capable	Limited reach and loss-to-follow up of target population for profiling and coaching, causing low confidence in own capabilities; and hindering further development of essential competences
		Other personal attributes	Strong ‘basic profile’ of nurses’ learning capacity and (potential) competence	Poor involvement and interest of other team members (especially physicians), diminishing nurses’ motivation
			Visible results and progress on lifestyle, wellbeing, risk perception in participants, boosting nurses’ motivation	Limited reach and loss-to-follow up of participants for profiling and coaching, diminishing nurses’ motivation
				Pitfall of health care providers to taking the lead hinders fidelity to patient-centered approach
	Process	Planning	Recognizing the time that is needed for the project and drafting a feasible plan; re-evaluating and adapting this plan along the way	Lack of dedicated time for implementers to carry out intervention components; due to low relative priority of the implementation
		Executing	Appointing internal practice manager, coordinating project activities	Lack dedicated time for central coordination of the intervention among other practice activities
			Nurses’ ability to use clinical judgment in profiling and coaching within setting of general practice	Lack of overarching internal protocols for management and follow-up of participants for cardiovascular disease, resulting in discontinuity in care
				Insufficient description of physicians’ roles (e.g., high-risk group)
			Support from project team: easily accessible, personal contact, understanding and knowledgeable, participator approach to overcoming barriers, flexible	
			Executing intervention components on a regular basis, with balanced participant flow and intensity ensuring progress on several aspects	
		Reflecting and evaluating	(re-)Defining roles and responsibilities along the way, reflecting on project status and adjusting goals and processes in internal team meetings and with project team	

**Table 3.** Examples of how barriers were addressed along the process; translated into implementation strategies^*^ and actions related to RE-AIM dimensions

Finding	Implementation strategy	Action
Reach		
Participant recruitment strategies have limited effect on reach; difficulties in reaching vulnerable target population	Adapt and tailor to context	The project team promoted adaptability of recruitment strategies; for example using flu vaccination campaign as entry point to invite eligible participants to increase reach; developing setting-specific information poster to better inform and activate the target population; engaging other team members such as receptionist for a low threshold and personal approach.
	Use evaluative and iterative strategies	Project team and implementers obtained and used participant’s feedback on facilitators and barriers they experienced by semi-structured telephone interviews and informal dialog. Participant’s feedback was implemented; for example emphasizing (health) benefits, giving small stepwise parts of essential information.
	Support implementers	Together with the implementers, the project team developed information sheets to be placed on desks in physicians’ offices, to remind them about the project and help them recall essential information about it, and to prompt them to actively recruit eligible participants.
	Develop stakeholder interrelationships	The project team captured good practices and local knowledge on strategies that work from implementation settings and shared it with the other sites to be contextualized and scaled up, for example, information sheet (implementers) and poster (target population) and case finding strategies.
Adoption		
Adoption is hindered by the intensity of intervention and variable COVID-19-related workload; adoption differed between implementers in general practices	Adapt and tailor to context	The project team promoted adaptability by giving implementers the opportunity to tailor frequency of the coaching sessions to the needs and preferences of participants. Also, implementers could define periods of decreased participant inclusion in order to be responsive to the context of the pandemic and still be able to guarantee high quality of care.
	Develop stakeholder interrelationships	The project team worked closely together with champions and early adopters in each general practice; for example nurse, general practitioner, student intern; to learn from their experiences and to disseminate those among other team members, using pre-existing communication channels such as team meetings.
Implementation		
Problems related to technicalities and project tools; low self-efficacy of implementers; insufficient collaboration with community resources; and aspects of implementer roles and responsibilities impede implementation	Provide interactive assistance	The project team facilitated implementation by introducing weekly informal contacts with key implementers from each setting for interactive problem-solving, responsive troubleshooting and vital support. The project team appointed members to offer local technical support for electronic data capture system and other tools. The project team also facilitated use of community resources by providing a basic overview of initiatives in the neighborhood of each setting and providing assistance and advice from an expert in the field to find care partners to answer specific participant needs.
	Use evaluative and iterative strategies	The project team conducted a needs assessment to identify gaps in knowledge and skills of implementers; process bottlenecks and emergent or potential problems to gain insight in the support that was needed.
	Train and educate implementers	In response to the implementers’ needs, the project team: - Conducted ongoing (refresher) training on all intervention components. - Developed and distributed educational materials to all implementers by different means, for example risk profiling and risk communication guidebook; behavior change counseling manuals to guide nurses’ coaching sessions, and to inform physicians and other implementers. - Introduced dynamic elements to the basic training (e.g., role play) - Used train-the-trainer strategies in collaboration with an expert center, so that implementers acquired skills to guide other team members. - Created a learning collaborative by organizing expert-led supervision sessions for nurses of the same practice. The session included feedback on nurses’ performance and tools for intervision so that they could further develop their competences. - Worked with educational institutions and expert organizations to develop evidence-based educative materials of high quality.
	Develop stakeholder interrelationships	The project team captured good practices and local knowledge on implementation and shared it with the other sites to be contextualized and scaled up; for example the benefits of a central coordinating person (e.g., practice manager); advice on implementation and use of tools and materials; experiences with behavior change counseling expert supervision session. The project team promoted using internal communication networks to elicit ownership and discussion around project activities. The project team also identified a local implementer (e.g., practice manager) to be responsible for follow-up of project status; aligning project activities with existing workflows and systems; ensuring the implementation was on meeting agendas; stimulating evaluation and reflection within the team; coaching the nurses. The project team also promoted identifying and building networks in the community; for example by inviting (potential) care partners in the practice to get acquainted and discuss collaboration.
Maintenance Sustainable change requires alignment with local policy and incentives; structural educational support; supportive networks; but also compatibility with primary health care characteristics and target populations.	Develop stakeholder interrelationships	The project team created and engaged a ‘resonance group’ with macro-, meso-, and micro-level stakeholders that came together every few months to elicit recommendations for sustainability and maintenance.
	Train and educate stakeholders	The project team secured the sustainability and further dissemination of project tools and educational materials by making them available through the project’s website. The team also engaged the regional postgraduate education ‘Nursing in the general practice’ to embed essential elements of the developed training in their curriculum. Moreover, the project team also organized several educational meetings with local associations for physicians and community partners.

* Adapted from Powell *et al.* (2015) and Waltz *et al.* (2015) [1,2].

### Reach

Personal invitation during a consultation appeared to be the best strategy to engage the target population; a strategy that was scaled up to all settings, reinforced by a poster design to inform and activate the target population.

‘For example, during our flu vaccination campaign. Most of the people we saw were eligible to participate. So we explained the project during the flu vaccination and we immediately received a lot of response’. (Nurse, Practice A)

Implementers described several factors that were taken into account when engaging people. In addition to the objective inclusion criteria, selection was also based on, for example, estimates of stage of change and the probability of effect.

‘If there are some psychological problems or they are having a hard time with something else at that moment, then I feel like that might not be the right time to open a conversation on prevention’. (Physician, Practice C)

For some of the implementers, the extent to which they felt competent also influenced the reach.

‘Certainly if they are people who have the tendency to “know better”, or already have their answer ready before you can propose something… I don’t want to coach such people, because it makes me feel so insecure. My knowledge is limited and then I come across as unprofessional’. (Nurse, Practice C)

Although active involvement of physicians in engaging the target population clearly improved reach, other priorities and insufficient involvement hindered adequate uptake of their role. The nurses developed information sheets to remind, inform, and activate the physcians as one of the actions to address this barrier.

‘I think the doctor can give some information, but I doubt if they truly familiar with all components of the project. We actually get very few patients referred. I think they just forget about it, they have a lot on their plate already during consultation’. (Nurse, Practice C)

The implementers felt the populations’s need for genuiness, authenticity, and active involvement of health care providers had become increasingly important during the COVID-19 pandemic. On the other hand, they felt that the pandemic has mainly had a negative effect on participation rates.

‘After the lockdown, we noticed that they are actually happy that they can come to us with their story, because we listen to them and show interest in their general well-being’. (Nurse, Practice B)

‘We actually see less people coming to the practice; out of fear of entering a contaminated environment … especially vulnerable people. Or fear of burdening us unnecessarily’. (Physician, Practice C)

### Adoption

The implementers indicated that the implementation climate in their setting was one of the determining factors for participation in the project. There was a very strong need for a more systematic approach to prevention.

‘Prevention must absolutely improve in primary health care. That’s a fact. I think we must play a more active role in it’. (Physician, Practice E)

The project’s intervention protocols and guidance were therefore seen as a major advantage for optimizing prevention in their practice.

‘I do think the project is very valuable. It gives us the chance to specifically focus on prevention… for the first time! And it also helps that we receive support and guidance’. (Nurse, Practice A)

At the same time, implementers indicated that change is needed in the currently limited task profile of the nurse. Implementation of the intervention was therefore seen as an excellent opportunity to explore further differentiation and expansion of the professional role of nurses.

‘I think it was a good first step for the nurses to take up new tasks. They felt the need to do more than only ‘the basics’ they were doing before’. (Manager, Practice A)

‘So many protocols have been written and yet nothing has actually changed so far. While us nurses were asking for new, challenging opportunities… I actually felt a bit useless here’. (Nurse, Practice C)

The complexity and intensity of intervention components, and the associated personnel resources, were mentioned as the main barriers to adoption. This is reinforced by the lack of a financial framework for prevention and interdisciplinary collaboration from the government, which was one of the main reasons for practice E to drop out of the study since they struggled with fitting in the project activities in their regular financial system. In response to intensity as a barrier, the settings altered participant recruitment activity to the dynamics of the COVID-19 pandemic. The resources required for project- and COVID-19-related activities could not be reconciled in Practice D; the main reason why this setting has also decided to discontinue study participation.

‘Because of the time investment… I just don’t think it is feasible in this setting. And it is not only the contact with the patient, but also the burden of questionnaires and administration’. (Nurse, Practice D)

‘The government should really be encouraged to better subsidize or finance such projects. Because we have to pay for our nurses ourselves and they can’t take on other tasks during project activities’. (Physician, Practice E)

When engaging implementers, it is important that everyone is involved from the start so that the project is supported by the entire team. Moreover, it is crucial that one or more people lead the implementation within the setting, according to our respondents. The local champions and early adopters in each setting shared their experiences with the project during team meetings in order to encourage team engagement.

‘Before a practice decides whether or not to get involved, it is important that everyone knows about it, and then collectively can decide whether or not they go for it together. Of course there must be a few team members really driving through the implementation’. (Physician, Practice D)

### Implementation

One of the key facilitators, mentioned by the implementers, was the adaptability of the project to each setting.

‘I think there was a lot of freedom to adapt everything to the context of our practice’. (Nurse, Practice B)

For example, the group with a high risk (red score) was also given the opportunity to participate in the coaching trajectory, after a shared decision with the nurse and physician.

‘Most people hope to get into the orange group for follow-up… they are even disappointed when they score red. So now we have decided that they can be followed up after we have consulted the doctor’. (Nurse, Practice A)

The COVID-19 pandemic was defined as one of the main barriers to the implementation.

‘We don’t know anymore… is it that we are structurally understaffed, or is it because of COVID-19. We are actually completely dependent of how the pandemic evolves, and it has a major impact on how we can plan our care and the project activities’. (Physician, Practice B)

Nurses felt that initially, physicians were not very involved, partly because the physician’s role was insufficiently clear. Implementers emphasized the importance of regular team meetings and discussion during the implementation process. Implementing the intervention has encouraged implementers to collaborate more closely in their settings, which can be facilitated by someone from the team who takes up a formal coordinating role.

‘I still miss the involvement of the doctors. I expected more feedback and more collaboration from them. I still think that the they don’t really know what is expected of them’. (Nurse, Practice A)

‘The communication in our practice has also improved as a result from implementing the intervention… because we need to discuss thinks like ’How is everything going?’ and ‘How can we do better’? We actually have to work together. We have to discuss together. We have to sit down together to see how we tackle barriers’. (Nurse, Practice C)

‘I think our practice manager has a good influence. Since she became more involved, she has proposed to bring the project on the agenda of our weekly team meeting’. (Physician, Practice A)

In all three settings, nurses have been given a more extensive and autonomous role within this prevention project. They proved to be crucial actors in the implementation.

‘I think the nurses have acquired a new role with this project. They now do part of the follow-up, which we normally did to a lesser extent. With this we were able to transfer an essential task. I think they are very suitable for this’. (Physician, Practice B)

It was seen as a major added value that nurses are able to carry out the project components from their expertise, clinical reasoning and within the medical context of a general practice.

‘We actually look beyond the profiling tool. Which makes sense, because we are trained to do so. We often measure blood pressure, or consult the patient record to see whether they take medication,… things like that’. (Nurse, Practice A)

Although they feel that the intervention machtes well with their competence profile, nurses emphasized the complexity of the coaching sessions with the aim of achieving behavioral change. After the first implementation round in Practice A, based on the nurses’ needs, the project’s training content and format were modified to increase proficiency in relevant competencies for their new role in behavior change counseling and scaled up as such in all settings.

‘Motivational interviewing… It’s difficult. I don’t really have much experience with that. With some of the participants you feel such resistance and a lack of motivation, and then I find it very difficult to get them to change their behaviour’. (Nursing assistant, Practice C)

Self-efficacy, job satisfaction, and motivation in nurses strongly depended on the results they do or do not achieve in the participants. They indicated that they needed confirmation of their abilities. In response to this need, the project team created a learning collaborative through expert-led supervision sessions where nurses received video feedback on their performance and tools for further intervision within their team.

‘I was able to give one patient a lot of information on healthy food, and he was completely open to that, while he usually is care refuser. So that went really well, and such “wins” give a lot of satisfaction’. (Nurse, Practice B)

The implementers also experienced the tension field between applying their own expertise and referring participants to community resources. The project team facilitated networking and making use of community resources, by providing assistance in navigating through the potential partnering initiatives and providers.

‘It is expected of us that we do everything ourselves. Both from the doctors and from the patients. But we aren’t specialists. We must indeed sometimes just refer people’. (Nurse, Practice C)

### Maintenance

Supplementary material 4 summarizes the components that the implementers intend to sustain the intervention, as well as the end user requirements to do so, linked to relevant CFIR domains and constructs. Implementers stated that the implementation process serves as a solid basis for continuing to develop and embed the general practice-level prevention policy in the future.

‘I notice that it has triggered something in our team, … We also want to do more than providing basic care and follow-up’. (Manager, Practice A)

The need for further reflection within the team was mentioned, to outline future prevention policies and to translate and tailor good practices from the project to sustainable action plans.

‘We will have to sit down together as team to see how we are going to proceed exactly. Are we only going to focus on disease prevention or more general health promotion? How are we going to invite the patients? Which profiling tool are we going to use?’ (Nurse, Practice A)

The implementers emphasized that sustaining the project requires close follow-up and communication in order to safeguard the continuation toward common goals.

‘I think we have really learnt from this project that we need to be more responsive in the future. In the beginning there were frustrations around the project, which were left unaddressed for too long. We need to communicate about this more quickly, sit together and look for solutions’. (Physician, Practice A)

The degree of compatibility with the current system and work processes also plays a major role to what extent this will be further embedded in general practice in the future, according to the implementers.

‘Prevention is just part of our responsibility, isn’t it. We certainly try, because we have the conditions to do it here too. We work with nurses, the doctors have a very clear vision, we work with a capitation system,…’ (Nurse, Practice B)

Implementers emphasized the tension field between the relative priority of prevention compared to other core tasks of general practice, which is strongly influenced by external factors. They mentioned that reorientation toward prevention requires investment in innovative capacity building of primary health care systems.

‘The general practice is consulted for all possible problems, which makes the workflow difficult to manage… You never know what the week is going to bring, and we have especially felt it with COVID-19. We urgently need to work on resilience of the system’. (Physician, Practice B)

According to the implementers, this is also possible through role expansion of interdisciplinary team work. The nurse in particular has proven to fulfil an essential role.

‘The project proofs that primary health care is broader than the general practitioner alone. What I especially learned from that… is that you can perfectly delegate prevention to the nurses. Even better’. (Physician, Practice A)

It was mentioned that there is a need for further consolidation of nursing roles through structural and ongoing growth and strengthening their competency profile. A crucial action we undertook was to engage relevant educational institutions to respond to this need.

‘As nurses become more involved in these kinds of processes, they should receive ongoing training, for example in intervision groups with others in similar trajectories’. (Physician, Practice B)

Additionally, they stressed the importance of a strong primary health care and welfare network with care partners to rely on for certain expertise. The project team reinforced this by resonating the findings in stakeholder meetings and educational meetings with local health care and community partners.

‘We have now seen how intensive this is. It is not possible for us to acquire all that knowledge, or to offer all that in our setting. So we need a strong network actually, in the region. The practice could take on a coordinating role’. (Physician, Practice C)

## Discussion

This paper describes the process evaluation of implementing a comprehensive program for the primary prevention of cardiovascular disease in five general practices in Belgium. We identified the factors that affected implementation success and sustainability and illustrated how barriers were addressed during the process by employing specific implementation strategies linked to concrete actions. Furthermore, we gained insight in the experiences of the primary health care teams with the implementation and examined nurse’s roles. These findings are meant to provide guidance for all relevant stakeholder groups that wish to scale up validated interventions for cardiovascular disease prevention in primary health care.

Several lessons have been learned during the implementation process. Foremost, the great potential of general practice as an important setting for primary prevention of cardiovascular disease, including risk profiling and lifestyle behaviour change counseling. This study especially highlighted the essential role of nurses in a transitioning primary health care toward health promotion and disease prevention and served as an opportunity to expand their scope of practice. Other studies show that nurses play a critical role in broadening, connecting, and coordinating primary and community care (Swanson *et al.*, 2020), by applying competencies such as patient advocacy, education, and people-centered care (World Health Organization, 2020). Recent evidence states that nurses have the extensive clinical experience to deliver major improvements in primary health care (Casey *et al.*, 2022). In various contexts, nurses increasingly and most effectively manage and coordinate care for people with, or at risk of, chronic disease, including tasks related to lifestyle risk counseling (James *et al*., 2019; Barr & Tsai, 2021). Despite competency potential to carry out intervention components, nurses initially felt underprepared, especially given the complex nature of behavior change interventions. Limitations of relevant competences have been previously identified as a barrier to nurses’ active involvement in preventive care (Volker *et al*., 2017). Our experiences are consistent with literature describing the need for ongoing education for upskilling existing nursing profiles to a more advanced level (James *et al*., 2019; Casey *et al.*, 2022; Morris *et al*., 2022), especially with regard to patient-centered communication (James *et al.*, 2020b), behavior change theories and counseling, and motivational interviewing; optimizing nurses’ effectiveness in communicating about lifestyle risk reduction and the reduction of chronic disease (James *et al*., 2020a; Hills *et al.*, 2022). Pioneering countries in integrating nurses in general practice, such as the United Kingdom, Australia, and Canada, demonstrate that introducing quality standards, linked with quality performance reimbursement, may support ongoing professionalization, unambiguous articulation of roles and scope, and development of formal educational and career pathways, hereby enabling nurses to practice to their full scope in primary health care teams (Parker *et al*., 2009; Halcomb *et al.*, 2017).

Second, this study highlights a number of barriers to reach vulnerable populations for prevention, despite the positive effects of combining engagement strategies. Reaching vulnerable populations for health promotion and prevention interventions is indeed challenging (Hoeck *et al*., 2014; Lim *et al*., 2019). When further scaling up similar preventive programs, more emphasis should be put on low-threshold approaches; population empowerment by enhancing health literacy; and social and health determinants of health care access. Our findings are supported by other research reporting on the promising context of primary health care to increase equity of health care access (Richard *et al*., 2016) and to decrease socioeconomic inequalities (Lorant *et al*., 2002; Meeus & Van Aubel, 2012).

In this study, our attempts to bolster collaborative action between general practice and community resources were limited to referral of participants to community resources, which were hindered by the lack of a strong linkage between primary health care and community organizations and lack of suitable community-led services. Our study shows the need for the currently fragmented landscape to shift toward integrated health care and welfare, by weaving networks with collaborative partnerships. In a related study within the SPICES project, which will be reported elsewhere, we also explored the opportunities of reaching vulnerable populations through existing community welfare organizations. In order to improve reach in future program planning and development, literature indeed recommends the integration of health and social care for vulnerable populations through multisectoral and community-based strategies (Richard *et al*., 2016; Corscadden *et al*., 2018). Previous studies have shown that this has great potential to increase community engagement levels and the reach of currently under-served populations, resulting in a positive impact on cardiovascular disease and its risk factors (Woringer *et al.*, 2017; Sidebottom *et al.*, 2021; Soltani *et al*., 2021).

Next, the lack of supportive financial and regulatory frameworks clarifying roles and shared responsibilities for interdisciplinary collaboration within primary health care teams were identified as main barriers to adoption. These findings are consistent with other studies describing the need for adequate funding, along with sufficient time and resources to facilitate the uptake of preventive actions in general practice and to mitigate the role constraints practitioners experience within current health systems (Volker *et al.*, 2017; Alageel *et al.*, 2018). Such support is also essential to enhance the continuity of preventive care and implementers’ commitment, confidence, and capacity to expand their scope of practice to systematically taking up preventive tasks (James *et al*., 2019; Morris *et al.*, 2022). In accordance with our insights stipulating the structural integration of health promotion and prevention into existing work processes and systems, evidence recommends policy makers to facilitate the delivery of such interventions during routine practice (Keyworth *et al*., 2020). Lastly, our study revealed characteristics of the implementation setting such as networks and communications, type of collaboration, and engagement of leaders as important influencing factors to implementer commitment and fidelity. Consistent with these insights, Russell *et al*. emphasized the importance of tailoring preventive interventions to practice size, implementer engagement and, especially the organisation of, and relationships between, the members of the primary health care team (Russell *et al*., 2019).

The COVID-19 pandemic has severely impacted the implementation in terms of increased workload; focus on acute care diminishing prevention; and avoidance of unnecessary patient contacts in the context of nonurgent care and disruption of health care planning. Our experiences are in line with a study exploring the impact of the pandemic on the core competences of primary health care. They reported that preventive care was compromised and chronic care was mostly postponed and raised concerns on the profound impact of the pandemic on health, and psychological and socioeconomic wellbeing in vulnerable populations (Verhoeven *et al*., 2020). In addition, COVID-19 patients with preexisting noncommunicable diseases are at higher risk of severe outcomes and mortality (Alzoughool *et al*., 2022). Many studies during the past few years have demonstrated the negative impact of the pandemic on lifestyle behaviors related noncommunicable diseases, such as increased snacking and alcohol consumption and consequently decreased adherence to healthy diets (González-Monroy *et al*., 2021; Bakaloudi *et al*., 2021b), higher incidence of overweight and obesity (Bakaloudi *et al.*, 2021a), and reduced physical activity and increases in sedentary time (Runacres *et al.*, 2021). It is clear that cardiovascular disease prevention should increasingly gain the attention of primary health care providers and policy makers in order to mitigate its burden especially in vulnerable populations. We therefore argue for reprioritizing health promotion activity within primary health care systems and for shifting toward a more preventive and integrated approach (Gibson *et al.*, 2022).

### Strengths and limitations

This is the first recent study that we are aware of to combine both RE-AIM-QuEST and CFIR frameworks to examine the implementation process of a complex multicomponent intervention in real life settings in a structured and systematic way. This approach enabled us to give a comprehensive insight into key factors, set out across the different CFIR domains and constructs, that can influence the reach, adoption, implementation, and maintenance of prevention programs in primary health care. Moreover, our flexible overall study design provided ‘actionable findings’ as defined by Keith *et al*. (2017) and valuable information and scope for adaptations that could be made to improve the uptake into general practice, through concrete actions addressing identified barriers across the various RE-AIM domains. This study therefore provides a practical example with broad application of how the complementary use of evaluation and explanatory frameworks, nested within a participatory action research design, can explain and improve implementation success and sustainability. Our study was further strengthened by the inclusion of all key implementers of the intervention in the different settings, and by the longitudinal evaluation during the implementation process. These methods have resulted in very rich qualitative data exposing the layered effort that is required to translate evidence-based preventive interventions into daily practice. Many of our findings as well as the used methodology could be of interest to research groups, policy makers, practitioners, and all those involved in implementing related health programs in similar contexts or those tackling the challenges related to transformations in primary health care. Transferability of our findings is further reinforced by in-depth description of our study context and the rigorous use of robust implementation frameworks.

Some limitations to this study should be considered when interpreting this work. One limitation relates to the timing of the post-implementation interviews which were intended to capture information on long-term sustainability. Since we were bound to the SPICES project’s time frame and planned the interviews shortly after the implementation period, we were only able to capture the end user requirements to realize their intention of sustaining the program. Finally, this study focused solely on implementer’s perspectives. We recognize the critical importance of the views and experiences of the vulnerable target population, as evidently they are directly affected by the integration of preventive interventions of novel nature into the services provided by their trusted general practice. We did in fact include patient participants to the profiling and/or coaching components in our project evaluation, but since this called for a different methodology, we have decided to describe these findings separately.

## Conclusions

The complementary use of RE-AIM QuEST and CFIR frameworks can be useful to guide the qualitative implementation process evaluation of a comprehensive intervention program for the primary prevention of cardiovascular disease in primary health care. General practice is an important setting for primary prevention of cardiovascular disease, and expanding nurse’s roles has great potential to build the capacity that is needed for scale-up and sustainability. Participatory strategies allow ongoing adaptation, enhancing uptake in practice. Actions related to adaptation to context, development of stakeholder interrelationships, and training and educating implementers are crucial to address barriers. Supportive financial and regulatory frameworks and a strong integrated community health model are needed to engage vulnerable populations and to increase long-term maintenance of prevention programs. Although COVID-19 has severely hindered implementation, our experience reinforces the urgency of health systems to shift toward a more health promotion and prevention-oriented care.

## Supplementary material

For supplementary material accompanying this paper visit http://doi.org/10.1017/S1463423623000063. click here to view supplementary material
