# Supplementary material for: Understanding factors affecting implementation success and sustainability of a comprehensive prevention program for cardiovascular disease in primary health care: a qualitative process evaluation study combining RE-AIM and CFIR
Source: Prim Health Care Res Dev. 2023 Mar 8;24:e17. doi: 10.1017/S1463423623000063 (PMC10050826; doi:10.1017/S1463423623000063)
Supplement: Supplementary file 1 [file phcsup.zip › S1463423623000063sup001.docx]

**Supplementary File 4: Factors influencing intention to maintain intervention program components**

Outer setting

External Policies and incentives

- Prevention should receive more media attention, e.g. in a government campaign, to increase ownership in the population

Sustainable implementation
& maintenance of primary prevention programs for cardiovascular disease in primary health care

Readiness for implementation

- Project tools/materials for dissemination need to be easily accessible and user-friendly
- Resilience of the inner system needs to be strengthened through e.g. team composition and resources (nurses are essential)

Process

Planning Engaging Executing Reflecting & evaluating

Structured and project-based approach; Combination of tailored strategies Structural incorporation of prevention; Long-term monitoring to detect

Translating plans into goals and actions to reach target population; Ensuring dedicated time and resources; population- and practice- level benefits;

adapted to available resources Creating ownership in all Shared responsibility of the team; Continuous and iterative consideration

team members Structural coordination and follow-up and adaptation to dynamic context

Networks and communications: Communication, collaboration and participation by all team members, working towards shared goals, supported by mission and vision, is necessary

Implementation climate

- Sustainable implementation in existing workflows and systems determined by compatibility, e.g. priority of prevention in practice vision, supportive financial structure, interdisciplinary collaboration forms; communication and continuity supported by electronic health record

- Need for continuous learning requires development of ‘lifelong learning’ support; e.g. further training, supervision and intervision cross-setting within learning community

Self-efficacy, competence and motivation

- Strong need for further consolidation of nurse’s role and related competency profile e.g. behavior change counselling techniques, empowering vulnerable people

- Healthcare funding should give more emphasis on preventive care, especially in the light of COVID-19

- A strong prevention framework is needed to support nurses and implementers

[Target population needs and resources](https://cfirguide.org/constructs/patient-needs-and-resources/)

- Target population, especially vulnerable people, should be empowered to become active partners in health e.g. by increasing health literacy

Cosmopolitanism

- Fragmented landscape needs strong integrated health care and welfare network with collaborative partnerships

Characteristics of implementers

Inner setting
