# Supplementary material for: Understanding factors affecting implementation success and sustainability of a comprehensive prevention program for cardiovascular disease in primary health care: a qualitative process evaluation study combining RE-AIM and CFIR
Source: Prim Health Care Res Dev. 2023 Mar 8;24:e17. doi: 10.1017/S1463423623000063 (PMC10050826; doi:10.1017/S1463423623000063)
Supplement: Supplementary file 1 [file phcsup.zip › S1463423623000063sup002.docx]

**Supplementary File 2: Topic guides used for individual and small group interviews with implementers**

# Structure and aims for each topic guide

**1. PRE-IMPLEMENTATION interview**

Timing: Before the start of Profiling/coaching

1. PART 1 Contextual analysis: The purpose of this interview is to describe the context in which the project is initiated. The specific setting (general practice) and the context (initiatives around prevention and healthy lifestyle from within general practice and in interaction with the community) are examined and described here by the researcher.
2. PART 2 Planning: The purpose of this interview is to describe the planning of the intervention program in the specific setting. The researcher will focus more on concrete planning of the implementation, in order to be able to adapt the intervention program components as well as possible to the needs and preferences of the general practice.

**2. PER-IMPLEMENTATION interview**

Timing: Is the guiding principle here. At the request of the implementers (perhaps especially nurses) this may be needed more quickly/more often in order to be able to intervene quickly on problems, needs.

- Within 1 month after the start of profiling
- Within 1 month after the start of coaching
- Within 2 months after the start of the project
- Within 4 months after the start of the project
- Within 6 months after the start of the project

During implementation, the process is monitored, so that implementation outcomes can be explained. The ‘plan, do; study, act’ cycle will be used here. The aim of these interviews is always to get an update on the progress, the process, the implementation and to be able to intervene quickly on problems and needs; and possibly adapt the intervention/implementation strategies accordingly.

**3. POST-IMPLEMENTATION interview**

Timing: Between 11 and 13 months after the start of the project

The purpose of the interview in this phase is to visualize how implementers look back on their participation in the project, to look back at the entire process, to ask to what extent the project (or its components) will be embedded in regular practice and which are the most important take-home messages for further roll-out in other settings in the future.

**4. EXIT INTERVIEW**

An important aim of our research is to be able to frame these concerns and systematically include them in our analysis & decision, thus capturing data from settings that decide to stop the implementation is also very valuable as a result. The purpose of this exit interview is to zoom in more deeply on the strengths and weaknesses of the project (within the broader context). This is a learning experience for the project team to take into account when scaling up to settings.

# Guidebook

1. Informed consent to participate in the study and publish results
2. Introductions
3. Outline the research theme and objective
4. Indicate the method of data collection and go over the course of the interview
5. Practical agreements before starting the interview
6. Interview or focus group discussion
7. Close interview, explore snow balling other key stakeholders and interest in member checking and/or participation in the implementation study

PRE-implementation interview

## PART 1: Contextual analysis

**Topic 1: Description of the organization/setting (general practice or welfare organization) –services, capacity, population**

*Description and aim:* Learn about different aspects of the context. Describe the shared vision and mission by which team members are bound, structural layout, organization of work process, communication channels, existing care/welfare partnerships and team composition. Take notes on financial structure, services and capacity. Describe what vulnerability means in the organization; which vulnerable subpopulation the organization works with and their characteristics, needs and expectations. What are ways to effectively reach and activate this population for prevention; i.e. channels, initiatives, communication.

Setting characteristics

- How does this organization work?
- Describe the context: professional relationships, leadership, communication channels, etc.?
- How long has setting been around? History?
- What types of patient populations are reached in your setting?
- Who are the members of this team?
- What are the mission and vision of this setting? How were they developed? To what extent do the members of the team agree with it? How important is it?

Population characteristics

- How would you describe the population in your setting?
- Describe the meaning of vulnerability in your setting
- For which subpopulation would cardiovascular disease risk assessment and monitoring of lifestyle behavior be extra important?
  - What needs does this population have?
  - How do you think we can best reach this target group?

**Topic 2: Past, current or planned practices in cardiovascular disease prevention and health/wellbeing related topics**

*Description and aim:* Define the working topics with regards to health/wellbeing this organization had experience with. Outline the current organization regarding prevention and describe to what extent are past/currently/future initiatives focused on prevention. Learn about the procedures, guidelines this was based on and what positive and negative experiences, gaps, possibilities for improvement, barriers or facilitators are. What are the experiences with risk assessment & communication and lifestyle coaching and what does the organization need to achieve goals.

- - - In general, which aspects of prevention are currently rolled out?
      - Mainly primary or secondary prevention?
      - Emphasis on certain conditions?
    - Specific for cardiovascular disease (primary/secondary)
      - Is anything currently embedded in practice? Describe the components and processes.
      - Initiatives around a healthy lifestyle? Describe the components and processes.
      - What are the gaps, barriers?
      - Would you be willing/able to do more around health and prevention? What could help you with this, what do you need to achieve this?
    - Role definition of all care providers involved in preventive actions
      - What disciplines are involved to start the prevention program/initiatives? Who is responsible for what?
      - Which competences would be needed to carry out risk assessment and communication or to work with people on behavioral change?
      - How do you (try to) achieve behavioral change in people?
      - Are there people in your organization who have received additional training in prevention or are there experts in this area? Who are these people and what training have they followed exactly?

**Topic 3: Link between primary health care and communities**

*Description and aim:* Describe the interaction between primary health care and communities. Describe the link and referral processes to community initiatives around prevention. Reflect on the paradigm shift from health and wellbeing around prevention. Outline key figures or instances, collaborations, networks and communication channels between organizations (cross-setting, cross-level).

- To what extent do you have relationships/networks within this organization with colleagues/people from similar organizations outside your own setting, e.g. describe other general practices, welfare organizations?
- What kind of information exchange is currently taking place between you and these other organizations outside your own setting?
- Which initiatives, related to the topic of the intervention program, are you familiar with? Specifically on primary prevention of cardiovascular diseases within the Antwerp region?
  - - To what extent are you aware of the “offer” in the community and to what extent are you “visible” in the neighborhood as organization?
    - To what extent is there currently interaction with the community? How do you collaborate with organizations, initiatives, key figures and how do you refer the population?

**Topic 4: Partnership and attitude towards intervention program implementation project**

*Description and aim:* Explore general attitude, trust and confidence towards the intervention program. Describe to what extent the intervention program would fit with the organization in terms of culture, beliefs, values and norms, mission and vision. Is intervention program in line with the organization objectives, needs and preferences and what is its relevance or relative priority. Explore how intervention program would be integrated into existing work processes and practices. Describe the feasibility of the intervention program in the context and setting and possible ways to adjust the project to the organization. Reflect on the anticipated impact of intervention program on the organization and its target population with regard to (dis)advantages and value in comparison to regular practice. Define to what extent this might require to collaborate in new ways within the organization members. Is participant open to acknowledge role expansion of the organization and in what circumstances would stakeholders benefit most? Who is or could be involved (practice nurse, lay counsellors) and identify competences and skills, and training needs.

We are now planning implementation of this project in the Belgian context.

- What do you think is important and meaningful?
- Can you think of barriers and facilitators to implementation?
- What do you think is feasible?
- What do you think is acceptable?
- What do you think is appropriate?
- What needs to happen or change for you to be able to speak of a positive evolution in the situation regarding the accessibility of cardiovascular disease prevention interventions for vulnerable target groups?
- What could be your input? (= concrete questions for cooperation)
- What would you need as support in your context? What support would be needed and what needs would your organization have?

## PART 2: Planning

**EXTRA TOPIC INTERVENTION PROGRAM during and after COVID-19 pandemic**

Due to the current COVID-19 crisis, all research activities for intervention program were temporarily suspended. Since it is not clear when we will be able to implement the already discussed interventions as planned in your setting, we invite you to think creatively and innovatively about alternatives with us. In this way, we also want to respond to contemporary challenges within the project, without losing sight of the importance of the prevention of cardiovascular diseases, which is certainly more important than ever in this situation.

- Now during the crisis, but possibly also afterwards, where are the priorities within your setting and what is essential for you in practice?
- What support for cardiovascular disease prevention, risk assessment and/or coaching for a healthy lifestyle could you use during or after the corona crisis?

The project team is considering to develop an online/virtual program for risk assessment and communication, as well as coaching people with moderately increased risk. We provide technical support (e.g. making Interheart tool available online). The training we provide for profiler/coach would therefore also take place online.

- What do you think of making these things available online? Do you think this is feasible?
- How do you see this in practice
- How could we reach people to participate in the profiling?
- To what extent do you think it would be feasible (and in what way) to adapt the coaching trajectory of people with moderate risk to the current context?
- How would we best tailor our training for both profiling and coaching to this?

**Topic 1: Embedding intervention program in practice**

- What was the most important determining factor for you to be convinced to participate in this project?
- How do you think the culture within this organization (general beliefs, values and norms, mission and vision, assumptions) will influence the implementation of the intervention program?
- To what extent is the implementation of the intervention program in line with other objectives within the organization?
- To what extent does the intervention program fit in with existing work processes and practices within your setting?
- To what extent and in what ways do you think the intervention program will meet the needs of your target population?

**Topic 2: Ideas on the anticipated barriers and facilitators**

- Describe the feasibility of the intervention program in your context and setting.
- To what extent will it be necessary (and are you open to it) to collaborate in new ways within your team and beyond, to implement intervention program?
- What changes or adjustments do you think are needed to adapt the intervention program so that it will work in an effective way in your setting?
- Who are the most important, most influential key figures in your setting to participate in this project?
- How will your organization's infrastructure (social being, age, maturity, size, physical layout) affect implementation?

**Topic 3: Anticipated “impact”**

Compare the intervention program with regular care in your setting.

- How does the intervention program differ from the usual way of working?
- How will it affect the nature of your work?
- What advantages/potential added value does the project offer compared to existing initiatives in the setting?
- What disadvantages does the project have compared to existing initiatives in the setting?
- How important is this project in meeting the needs of your organization's target population, or other objectives in the setting?

# PER-implementation interview

**Topic 1: Experiences, barriers and facilitators**

- Describe the progress so far of the intervention program in your setting. What is your overall impression?
- Which characteristics of the context in which you work influence or determine the implementation of the intervention program and how do you deal with this?
- To what extent do all team members within the general practice participate in the different components of the intervention program?
- What is your experience and feeling about your new role within the intervention program: as a profiler, coach and/or implementer?
- What knowledge and skills have you acquired and which competencies need to be further developed?
- What else would you like or need to strengthen your competencies?
- What is your perception of the quality of the project as a whole, the different components and the supporting materials/tools/training?

**Topic 2: Ideas and perception about the current impact**

- What is your general impression on the impact of the project at the moment?
  - - - On how it works in practice
      - To you and your colleagues
      - On the target population
- To what extent are the implemented components of the intervention program evaluated?
  - - - Is time and space made available for reflection? (e.g. does this come back to meetings?)
      - How is this dealt with in practice? Who is involved in this and how are you going to get started?

**Topic 3: Follow-up of the planning of intervention program components in practice**

ADJUST or CHANGE and HOW GOOD:

- - - Which components of the project do you think should be tailored or personalized OR have you already adapted in the meantime? Describe what, why, when and how.
    - Which components of the project do you think should be changed OR have you already adapted? Describe what, why, when and how.
    - To what extent was the intervention carried out according to the planned schedule? Who monitored this? If strategies were used to maintain or improve fidelity, describe them. How can this be improved in the future?

# POST-implementation interview

**Topic 1: End product INTERVENTION PROGRAM implementation**

- What do the different components of the intervention program look like in your setting after one year? Describe.

**Topic 2: Long-term sustainability and maintenance**

- Which components of the intervention program have been preserved so far?
  - - - What do you think is unnecessary to keep and why?
      - What do you think is most valuable to keep and why?
- How is the continuation of the implementation of the intervention program (or parts of it) in your own setting planned?
  - - - To what extent do you plan to integrate which components in future practice (after the project has ended)?
      - Who will remain/become involved in this, who do you need for this?
      - What are the barriers and facilitators to maintain this intervention program in the longer term and how can these be tackled or strengthened?
      - What costs will you have to consider when you decide to continue the project (or components of it)?
      - To what extent can you integrate the intervention program into your professional mission and (legal) responsibility/professional domain?
      - What kind of support (structural, financial, other incentives) could influence your decision or willingness to continue the intervention program?
      - What resources do you need to continue the project and get the different components to the target population, and are they sufficient?
- What do you think are the crucial components of the intervention program for the provision of care to your target population and as an aspect within your practice, and which characteristics should definitely be adopted in a next setting?
  - - - What lessons can be learned from this project if implemented in a similar context?
      - How should this be done in order to support this as well as possible?

# EXIT interview

**Topic 1: 'icebreaker'**

The aim is to start a conversation in a safe environment.
Have their role described in the preparation and planning of the implementation of the intervention program

- - - To start with, I would like to go back to how you got to know the project?
    - What was your first impression when introducing the intervention program in general?
    - We have already started preparations for the implementation of the intervention program within this practice. What were your experiences?

**Topic 2: INTERVENTION PROGRAM in specific setting**

The aim here is to get a better view of the implementation process within the setting, and especially what happened behind the scenes. Also briefly evaluate the training.
Briefly describe in this topic which steps have been taken in their perception to introduce and plan the project in this setting

I understand that … was the first to be introduced to the project.

- - - How was this further communicated/proposed in practice?
    - To what extent did the project “live” in your practice?
    - Were other colleagues besides the doctors/nurses involved?
    - What was your experience with the communication with the project team?

Engagement for collaboration with the project.

- Was it agreed in practice who would mainly be involved in this project?
- What convinced you to participate in the project?
  - Did you all share the same opinion on this?
  - How long did it take for you to agree on this decision?
  - What did you think were the strengths of the project? What should definitely be preserved?

Preparations for implementation.

- How did you feel the preparations for the intervention program went?
- What is your perception of the quality of the project as a whole, the different components and the supporting materials/tools/training?
- What did you think of the training you already attended?

**Topic 3: Explore the decision to drop out the project**

The aim here is mainly to gain more insight into the reasons for withdrawing from the project, the motivation, strengths and weaknesses, what lessons can we draw from this?

Concerns have been raised at some point, let them describe the moment when they first came up.

- At some point, concerns were expressed to us that played within your practice. What was going on?
- Who raised these concerns and to what extent did this influence the vision of the entire team?
- What did the COVID-19 pandemic bring as an obstacle? Was a solution quickly found for this? How did you deal with this? How would the patient deal with this?

Possibly ask questions if this is mentioned: culture within the setting, place of intervention program in relation to other objectives (priority/added value), place of intervention program within a certain workflow and structure

- What are the barriers and facilitators to maintain this intervention program in the longer term and how can these be tackled or strengthened?
- Which do you think are the crucial components of the intervention program for the provision of care to your target population and within your practice, and which characteristics should definitely be adopted in other settings?
- What lessons can be learned if we want to start up in a similar context? What can we do differently/better in the future?
