# Supplementary material for: Understanding factors affecting implementation success and sustainability of a comprehensive prevention program for cardiovascular disease in primary health care: a qualitative process evaluation study combining RE-AIM and CFIR
Source: Prim Health Care Res Dev. 2023 Mar 8;24:e17. doi: 10.1017/S1463423623000063 (PMC10050826; doi:10.1017/S1463423623000063)
Supplement: Supplementary file 1 [file phcsup.zip › S1463423623000063sup003.docx]

**Supplementary File 1: TIDieR checklist with description of the SPICES implementation program**

| 1. Brief name | The evidence-based SPICES program  Note: SPICES stands for ‘Scaling-up Packages of Interventions for Cardiovascular disease prevention in selected sites in Europe and Sub-Saharan Africa’. |
| --- | --- |
| 2. Why | The burden of Cardiovascular diseases can be reduced by targeting lifestyle determinants such as physical inactivity, unhealthy dietary habits, smoking and excessive alcohol intake. Likewise, knowledge of behavioral risks is the central element of lifestyle change and individuals who perceive themselves at higher risk of cardiovascular disease are more likely to adopt a healthy lifestyle. Interventions on risk profiling and lifestyle coaching can raise awareness on the individual risk and may have positive effects on risk perception, increase a participant’s knowledge and skills to reduce the individual risk, and improve healthy lifestyle behaviors including healthy diet, physical activity, smoking cessation and reduction of alcohol consumption. By improving modifiable risk factors, the individual risk will decrease. A combined approach of community- and primary care-based implementation of the interventions aims to lead to higher reach of (vulnerable) target populations and increase the uptake of interventions. |
| **WHAT** |  |
| 3. Materials | **Training of implementers**  We developed training manuals and dynamic training modules with role-play; refresher trainings and supervision sessions with expert video-feedback to support and strengthen the competences of the implementers carrying out the profiling and coaching interventions.  For all implementers, the training content consisted of several elements: basic information of aims of the SPICES project; reaching and engaging (vulnerable) target populations; profiling scenario (semi-structured guidebook model-sentences); use of devices and Redcap software/tools for data collection; risk communication techniques including self-perception; risk category; reflection; translating lifestyle advice; coping with resistance; follow-up; and referral.  Implementers carrying out the lifestyle coaching component received more in-depth training on motivational interviewing techniques, behavior change models and techniques, and communication and interaction with vulnerable groups.  **Informative invitation leaflet/poster**  In the general practice: Provided in waiting room and general practitioner (physician) and practice nurse office, website and context-specific communication channels  In community settings: Provided in communal spaces, local newsletter, website and context-specific communication channels  **Guidebook for participant invitation & risk communication**  Implementers carrying out the profiling component received a semi-structured guidebook including model-sentences (profiling scenario) to guide the participant invitation and engagement process, and to support the risk profiling, risk communication, tailoring the lifestyle advice, and initiating the appropriate follow-up trajectory.  **INTERHEART risk score translated & contextualized**  Implementers carrying out the risk profiling, used the non-laboratory INTERHEART risk score. The INTERHEART Risk Score is a validated score that includes data on age; sex; status with respect to smoking, diabetes, high blood pressure, and family history of heart disease; waist-to-hip ratio; psychosocial factors; diet; and physical activity. The tool was made available in Redcap for use online on personal computer or tablet.  **Risk cards**  Implementers carrying out the profiling are supported with ‘Risk Cards’ developed for each risk category, including an explanation of the meaning of individual risk, a visual representation of the individual risk score, and a narrative.  **Lifestyle plan**  Implementers carrying out the coaching are supported with a ‘Lifestyle plan’ to guide their coaching sessions, in which behavior change techniques were also integrated. Based on brief action planning for health.  **Follow-up questionnaires**  Part of the intervention since implementers used this information to further explore the participant’s lifestyle behaviors and risk perception during the coaching sessions.   - **ABCD questionnaire**: cardiovascular disease risk perception - **Short IPAQ**: Activity level using the shortened version of the International Physical Activity questionnaire (validated in Dutch) - Improvement of diet & alcohol: **DASH-Q** (Dietary Approaches to Stop Hypertension) + added questions from **Feel4Diabetes diet questionnaire** (validated in Dutch) & contextually adapted to Health Promotion Institution Flemish Government recommendations.   **Data collection tablets**  We provided the implementers carrying out data collection during profiling and/or coaching with tablets to support them in mobile data collection on location.  **Training videos**  In collaboration with a local fitness center, the team developed five 35-minute, moderate-to-high intensity work-out videos with minimum impact on joints (using a chair), that were made available online for implementers to refer eligible participants to.  **SPICES promotion materials**  With the intension of increasing visibility of the SPICES project in partner organizations, we designed T-shirts with a brief motivational quote (“Prevent cardiovascular disease, live healthy!”), the SPICES logo and the logos of the funding body and research group institution. T-shirts were worn during training activities and profiling and coaching activities. We also designed a banner to set up at any event to recruit/engage/inform the target group and stakeholders.  The materials are in Dutch and are available upon request from the SPICES research team. |
| 4. Procedures | **Participant recruitment**  The target population are adults between 40-75 years old, who did not have an event before, of all socio-economic categories, but with extra effort to reach those with low socio-economic status. At general practice level, the strategies used to inform, invite and engage the target population differ in each setting. Examples of participant recruitment are: personal invitation by practice nurse or general practitioner during a consultation; personal invitation by practice nurse or general practitioner during the flue vaccination campaign; extracting the target populations from the patient records and inviting participants through email or telephone. After giving potential participants information about the project, they were invited to make an appointment with the practice nurse for risk profiling. The implementers made use of informational pamphlets and posters in the waiting room and in the office.  **Profiling**  Profiling took place with the aid of the INTERHEART risk score. Based on this profiling tool, participants are divided into three groups: green (low risk), orange (intermediate risk) or red (high risk). An automated lifestyle advice gets generated by the system and is tailored to the individual answers. The result is communicated with the aid of risk cards. Based on their individual cardiovascular disease risk, the appropriate follow-up trajectory is proposed in a motivational manner: Everyone gets brief advice on basic recommendations for a healthy lifestyle, the red group is referred to usual care, and the orange group is invited to participate in the lifestyle coaching sessions.  **Coaching**  The lifestyle coaching sessions are focused on raising awareness of individual risk and modifiable risk factors related to lifestyle (diet, physical activity, smoking). With the aid of the lifestyle plan, the participant and the coach work together towards behavior change. Several behavior change techniques are embedded within the coaching; goal setting, action planning, problem-solving and motivational interviewing. Implementers are supported to refer participants to existing community resources. |
| 5. Who provided | In **primary care settings**, profiling and coaching were carried out by the practice nurse (and one nurse assistant) within the general practice team. All team members were involved in the project for informing and engaging the target population. In case of high-risk participants, a shared decision on the appropriate follow-up trajectory was made together with the practice nurses and the general practitioners. |
| 6. How | The intervention was delivered face-to-face in individual sessions. |
| 7. Where | In **primary care settings**, including five multidisciplinary general practices with capitation payment system, one of which was located in a rural area and four of which were located in vulnerable, urban districts in the city of Antwerp in Belgium. The intervention program was carried out in the implementers’ office. |
| 8. When and How much | The **profiling component** was delivered in one session. The duration is approximately 20 minutes.  The **coaching component** was delivered in 10 sessions, with set intervals and spread over 12 months. The duration is approximately 30 minutes. The follow up sessions (Sessions 1-10) were planned as follows:   - Session 1 - One week after profiling: Start up coaching with ‘Lifestyle Plan’ + Follow-up questionnaires   Month 1   - Session 2 – 14 days later (half month 1): Coaching + Did red group contact their physician yes or no (if no: reason why) - Session 3 – 14 days later (end month 1): Coaching   Month 2   - Session 4 - 14 days later (half month 2): Coaching - Session 5 - 14 days later (end month 2): Coaching   Month 3   - Session 6 – 4 weeks later (end month 3): Coaching   Month 4   - Session 7 – 4 weeks later (end month 4): Coaching + INTERHEART + Follow-up questionnaires   Months 5 & 6   - Session 8 – 2 months later (end month 6): Coaching   Months 7, 8 & 9   - Session 9 – 3 months later (end month 9): Coaching   Months 10, 11 & 12   - Session 10 – 3 months later (end month 12): Last closing session with long-term sustainable change planning + INTERHEART + Follow-up questionnaires |
| 9. Tailoring | We conducted a process evaluation with ‘plan, do, study, act’ cycles every two to three months during implementation. During this process evaluation, we also assessed the intervention components, the supporting project tools and the implementation strategies used. This entailed interviews and meetings with the implementers in each implementation phase and the co-creation and adaptation of the intervention components that are implemented. |
| 10. Modifications  (& rationale) | **COVID-19 related (temporary) modifications**   - During the first lockdown period (starting in March 2020) the SPICES project in Belgium moved the interventions online in May 2020 due to physical distancing recommendations during the COVID-19 pandemic lockdown. Participants were informed through the webpage of local partner organizations. Participants were then asked to complete the online profiling questionnaire. They received an information e-mail and a trained SPICES coach contacted them by phone to discuss their cardiovascular disease risk profile. - In the same period, we focused more on population level health promotion activities. In order to support vulnerable groups during this period, we developed messages with low-threshold advice and tips, based on existing messages from local public health organizations. The weekly messages were disseminated through the social media channels of our local partner organizations.   **Modifications in primary care settings**   - Participant recruitment strategies (as described in above in 4. Procedures) were tailored and adapted to the general practice needs and context. Practices shifted or adapted their strategies to increase or specify the reach of the target group. - The implementers suggested involving not only intermediate risk but also participants with high-risk score (red group) in the coaching session given that the participants are interested to participate. However, since this regards patients at high risk of cardiovascular disease, general practice teams are urged to develop an internal protocol or procedure for the evaluation of the individual patient situation (e.g. discussion between nurse and physician regarding medical background and medical treatment status, potential health benefit of participating in coaching traject, etc.) before considering the participant to be included in the red group follow-up. Such a procedure also ensures the patient safety and continuity of care for patients at high risk of cardiovascular disease and often with more elaborate medical background.   Note: Patients who already have a history of an event, are still excluded from the SPICES interventions (secondary prevention), since the intervention is exclusively developed for the primary prevention of cardiovascular disease. This is mainly done by adopting specific recruitment strategies.  Based on this rationale, the research team has decided to enrol red groups also to the coaching and follow up session only in the general practice setting. Hence, the SPICES project will have a follow up data of the participants with the red group (high risk score) in all of the settings. All the remaining project activities and the type of data will be as mentioned in the original intervention plan. - For participants who were either orange or red group and interested to participate in the coaching, the follow-up questionnaires on level of knowledge and perception of cardiovascular disease, physical activity, dietary history were collected either automated online surveys or face-to-face in the general practice prior to the coaching session. This was a modification that was made in one general practice, since the online approach would help the implementers to cope with any time restraints. - In some cases, the format and intensity of delivery of the interventions were tailored to the needs and preferences of the participant. This means that sometimes the coaching sessions would be held online in order to remove practical or contextual barriers. Also, the set intervals and number of coaching sessions could differ on the participant’s request, based on their individual needs and preferences, although the implementers always suggested the intervention as planned to be the best option. - Planning and follow-up tool: After implementation in 3 welfare organizations and before implementation in general practice, implementers raised the issue of the complexity of planning all coaching sessions. In order to assist the implementers carrying out the coaching, we developed a tool in Microsoft Excel for the practical planning of the 10 coaching sessions with set intervals, and for follow-up reports of the participant, including reminders for the coaches of tasks to complete in each coaching session. |
