# Supplementary material for: Understanding factors affecting implementation success and sustainability of a comprehensive prevention program for cardiovascular disease in primary health care: a qualitative process evaluation study combining RE-AIM and CFIR
Source: Prim Health Care Res Dev. 2023 Mar 8;24:e17. doi: 10.1017/S1463423623000063 (PMC10050826; doi:10.1017/S1463423623000063)
Supplement: Supplementary file 1 [file phcsup.zip › S1463423623000063sup004.docx]

**Supplementary File 3: Contextualized CFIR domains and constructs**

| Domain 1: Intervention Characteristics | This domain covers the questions whether the intervention program, consisting of 1) risk profiling and risk communication and 2) behavior change counseling for people at medium to high risk, is superior to the status quo and if it can be adapted to the current Belgian context. |
| --- | --- |
| Relative Advantage | The construct relative advantage was defined as the stakeholders’ perception of the advantage of implementing the intervention program versus regular care. |
| Adaptability and Trialability | The constructs of adaptability and trialability were often discussed together. Adaptability was defined as the degree to which the intervention program may be/should be/was adapted, tailored, refined, or reinvented to meet local needs. Trialability reflects the ability to test the intervention on a small scale in the organization, and then adapt certain components where needed. |
| Complexity | Complexity was defined as the perceived difficulty of the intervention program, reflected by duration, scope, radicalness, disruptiveness, centrality, and intricacy and number of steps required to implement. |
| Cost | Costs associated with implementing the intervention program including personnel cost. |
| Design Quality and Packaging | Perceived excellence in how the intervention program, including supporting tools and materials, is bundled, presented, and assembled. |
| Domain 2: Outer Setting | This domain describes the reasons why it is important to implement the intervention program in the current Belgian context, including gaps in patient care or regulatory conditions. |
| Target Population Needs and Resources | This construct entails defining the target population, and the extent to which the needs of the target population, as well as barriers and facilitators to meet those needs, are accurately known and prioritized. |
| Cosmopolitanism | This constructs reflects the degree to which organizations on primary care and community level are networked with other external organization and what the experiences are on existing collaboration and/or interaction and communication. |
| External Policies and Structures | This construct includes external strategies to sustainably implement and embed the intervention program, including policies and structures, as well as recommendations and guidelines. |
| Variable Factors | We included this construct for COVID-19- related findings. |
| Domain 3: Inner Setting | This domain covers the questions whether the intervention program fits into the target implementation settings (general practices) and to what extent it is feasible. |
| Structural Characteristics | The social architecture, age, maturity, and size (e.g. by number of multidisciplinary team members) of the general practice. |
| Networks and Communications | This constructs reflects the nature and quality of social networks and of formal and informal communications within the general practice. |
| Implementation Climate | The absorptive capacity for change, shared receptivity of potentially involved organizations to the intervention program, and the extent to which use of the intervention program is supported within eligible partner organizations. Aspects of three sub-constructs; tension for change; compatibility and relative priority, were discussed during the interviews. |
| Readiness for Implementation | The commitment of general practices to the implementation of the intervention program. |
| Domain 4: Characteristics of Individuals | This domain covers the questions whether implementers (general practitioners, practice nurses and other relevant team members in general practice) have the competences needed to deliver the intervention program. |
| Self-Efficacy | The construct reflects potential implementers’ individual belief in their own capabilities to execute courses of action to achieve implementation goals. |
| Other Personal Attributes | A broad construct to include other personal traits to describe the implementers’ profile; such as intellectual abilities, motivation, values, competences, capacity, and learning style. |
| Domain 5: Implementation Process | This domain covers the questions whose work is affected by the intervention program; how the intervention program can be best planned within a setting; whose input and expertise is needed; and how to engage implementers and the target population, in order to implement and sustain the intervention program. |
| Planning | The importance of developing a scheme or method of behavior and tasks in advance, in order for the implementation of the intervention program to be successful. |
| Engaging Participants | This construct concerns strategies to engage individuals served by the organization or the target population; i.e. patients in general practices. |
| Engaging Implementers | This construct concerns the process of attracting and involving appropriate implementers (i.e. team members from general practices or welfare organizations) that should/will be involved in the implementation of the intervention program in their setting; |
| Executing | This construct is about carrying out or accomplishing the implementation according to plan. This included consistency in performance and fidelity to the intervention program components. |
| Reflecting and Evaluating | This construct reflects the process of quantitative and qualitative feedback about implementation accompanied with regular personal and team debriefing and reflection about progress and experience from the implementers’ viewpoint. |
